# Association of gestational weight gain with adverse pregnancy outcomes across body mass index categories for twins in China

**DOI:** 10.3389/frph.2026.1752008

**Published:** 2026-05-14

**Authors:** Huiyuan Zhu, Xiaozhou Jia, Liqin Yang, Xianglian Peng, Kehan Zou, Yueyun Xiang, Donghua Xie

**Affiliations:** 1 Hunan Key Laboratory of Typical Environmental Pollution and Health Hazards of Hunan Province, School of Public Health, Hengyang Medical School, University of South China, Hengyang, Hunan, China; 2 Department of Obstetrics Ward II, Hunan Provincial Maternal and Child Health Care Hospital, Changsha, Hunan, China; 3 Department of Neonatology, Hunan Provincial Maternal and Child Health Care Hospital, Changsha, Hunan, China; 4 Department of Health Care Management, Hunan Provincial Maternal and Child Health Care Hospital, Changsha, Hunan, China; 5 Department of Clinical Laboratory, Hunan Provincial Maternal and Child Health Care Hospital, Changsha, Hunan, China

**Keywords:** adverse pregnancy outcomes, Chinese population, gestational weight gain, pre-pregnancy body mass index, twin pregnancy

## Abstract

**Objective:**

To examine the influence of Gestational Weight Gain on adverse pregnancy outcomes in twin pregnant women with different pre-pregnancy Body Mass Index Categories and provide more basis for prenatal guidance and monitoring of twins.

**Methods:**

Twin pregnant women with gestation age ≥ 28 weeks who had gave birth from 2022 to 2023 were considered as research subjects. Information on mothers and newborns was collected. Logistic regression model was used to estimate the independent impact of Gestational Weight Gain rate on the outcomes and the impact of Gestational Weight Gain rate on the outcome under different pre-pregnancy Body Mass Index Categories.

**Results:**

Overall, 919 twin pregnant women and 1838 newborns were included. The logistic regression model shown that inadequate Gestational Weight Gain in all groups significantly elevated the risk of preterm birth. Inadequate Gestational Weight Gain increased the likelihood of Small for Gestational Age infant (_a_OR = 2.10, 95%CI: 1.43–3.09). Besides, excessive Gestational Weight Gain raised the probability of Hypertensive Disorders of Pregnancy (_a_OR = 2.60, 95%CI:1.78–3.78) and Large for gestational age infant (_a_OR = 1.77, 95%CI: 1.24–2.51).

**Conclusion:**

In twin pregnancies, inappropriate weight gain might be correlated with preterm birth, Small for Gestational Age infant, hypertensive disorders of pregnancy and Large for Gestational Age infant. We propose that personalized counseling and guidance on weight gain and nutrition should be provided to women with twin pregnancies.

## Introduction

The advancement of Assisted Reproductive Technology (ART) in the last 40 years has contributed to a global increase in the rate of twin births (1). Twin pregnancies account for approximately 2%–4% of global births (2), the incidence of twin pregnancy in China is 3.3% (3). Compared to singleton pregnancies, the neonatal mortality rate for twins was significantly higher, showing a greater than sixfold increase (4) and an increased risk of preterm birth and complications (5), particularly in low- and middle-income countries (6). The Gestational Weight Gain (GWG) is widely recognized by experts in obstetrics and gynecology as one of the crucial and potential factors affecting the occurrence of pregnancy outcome.Previous researches have found that insufficient GWG led to an increased risk of preterm birth and Small for Gestational Age infant (SGA) (7, 8), excessive GWG was associated with an increased risk of Hypertensive Disorders of Pregnancy (HDP) and Large for Gestational Age infant (LGA) (9, 10).

Guidelines of GWG for singleton pregnancies cannot be applied to the twin population (11). The special physiological state of the increased plasma volume and placenta implantation means that twins need much more nutrition than Singletons. However, Guidelines of GWG for twin pregnancies were undeveloped. In 2009, the Institute of Medicine (IOM) proposed a recommended range for weight gain during pregnancy for twin pregnancies (12). Due to the limited sample size, this standard was considered only as an interim guideline. The data in studies on GWG in twin pregnancies were constrained by the limited number of twin births and the potential confounding effects of chorionicity and assisted reproduction, leading to inconsistent conclusions (13). For the population of twin-pregnant women in China, factors such as the lower body mass index (BMI) classification criteria for the Chinese population compared to those of the World Health Organization, as well as ethnic and dietary differences, make it difficult to directly apply the IOM guidelines to this specific group.

For twins prenatal guidance and monitoring to provide more evidence, this study aims to examine the individual and combined influence of GWG on adverse pregnancy outcomes, based on that suitable weight gain range of chinese twin pregnant women in.

## Methods

### Study participants

This is a retrospective cohort study, Twin pregnant women with gestation age ≥ 28 weeks who had gave birth in the Obstetrics and Gynecology of Hunan Maternal and Child Health Hospital from 2022 to 2023 were considered as research subjects. Using the electronic medical record system, we have gathered the following information: maternal pre-pregnancy weight, height, maternal age, past medical history, and Childbearing history, chorionicity, mode of conception, gestational age, pregnancy complications, birth weight and short-term neonatal complications. The exclusion criteria were set as follows: (1) Twin to Twin Transfusion Syndrome (TTTS); (2) Neonatal death caused by embryo reduction, induced abortion, or other reasons; (3) Newborn malformation or unknown outcome; (4) Basic information of pregnant women unknown; (5) weight gain during pregnancy not reported or logically incorrect; (6) monochorionic monoamniotic twins (MCMA); (7) The pregnant woman had diabetes, hypertension and thyroid diseases before pregnancy. This study involves human participants and was based on the Declaration of Helsinki. This study was approved by the Ethics Committee of Hunan Provincial Maternal and Child Health Care Hospital (NO. 202304) on February 22, 2023.

Chorionicity was identified by ultrasound examination at 8–12 weeks of gestation. The gestational week was determined by the last menstruation and ultrasound examination. If assisted reproductive technology was utilized for conception, the first day of the last menstruation is considered to be 17 days from the date of transfer for fresh blastocysts or 19 days for frozen blastocysts. The pre-pregnancy BMI of pregnant women were calculated as divide their pre-pregnancy weight (kilograms) by the square of their height (meters), the data of pre-pregnancy weight and height are measured by pregnant women under the guidance of medical staff. The prenatal weight is measured before delivery in hospital. According to Chinese standards (14), all pregnant women were classified into three categories: Underweight (<18.5 Kg/m^2^), Normal weight (18.5 ≤ BMI < 24.0 Kg/m^2^), Overweight (24.0 ≤ BMI < 28.0 Kg/m^2^). The study had a limited sample size of obese pregnant women (*n* = 15), who were categorized as overweight.

### Exposure

The main exposure was total GWG, which was calculated as the difference between pre-pregnancy weight and prenatal weight. To avoid the impact of gestational length on total GWG, the GWG rate index was introduced, which is expressed as GWG rate = total GWG/gestational week, in units of kg/wk. Referencing to the research conducted by Li Gao et al. (15), the study identified the optimal weight gain rate range for pregnant women expecting twins (Underweight: 0.43–0.58 Kg/w, Normal weight: 0.41–0.58 Kg/w, Overweight: 0.35–0.55 Kg/w). Each pregnant woman's total GWG rate was explicitly classified as being below the recommended range (inadequate), within the recommended range (appropriate), or above the recommended range (excessive).

### Outcomes

Preterm birth gestational week less than 37, 36 and 34 weeks, Hypertensive Disorders of Pregnancy (HDP), Small for Gestational Age (SGA), Large for gestational age infant (LGA), Low Birth Weight (LBW) were the main outcomes of interest. Other outcomes included subclinical hypothyroidism during pregnancy, Gestational Diabetes Mellitus (GDM), Very Low Birth Weight (VLBW), neonatal hypoglycemia, Neonatal Respiratory Distress Syndrome (NRDS), neonatal sepsis, neonatal anemia and neonatal pneumonia were the outcomes of interest. According to the American Congress of Obstetricians and Gynecologists (ACOG) diagnostic criteria, “hypertensive disorder of pregnancy” was defined as the presence of at least one type of pregnancy-induced hypertension, preeclampsia, or eclampsia (16). According to the gestational age and gender of China twins' birth weight curves (17, 18), birth weight below the 10th percentile was regarded as SGA, and above the 90th percentile as LGA.

### Statistical analysis

The sample size was estimated using PASS 2021 based on comparisons of two independent proportions between GWG categories. A two-sided test with a significance level of α = 0.05, accounting for a 10% non-response rate and statistical power of 1−β = 0.80 was assumed. Separate calculations were performed for the normal vs. insufficient GWG groups and the normal vs. excessive GWG groups using incidence rates derived from previous studies (19, 20). The continuous variables were listed the average and compared using one-way analysis of variance (ANOVA), the categorical variables were represented by a ratio (%) and compared using the Chi-square test or Fisher exact test to compare maternal characteristics and outcomes across GWG rate groups. The GWG rate independent impact on outcomes and the GWG rate under different levels of BMI influence on outcomes were estimated with multivariate logistic regression model. For the evaluation of neonatal outcome, a logical model with generalized estimation equation (GEE) was used to solve the correlation between fetuses in pairs. Covariates included maternal age, first production, first gestation, chorionicity and mode of conception and gestation age.

In order to verify the robustness and reliability of the research conclusion, this study conducted two sensitivity analysis methods. First one, the core exposure variable was replaced by the total GWG, followed by the principal analysis and statistical method, to investigate the changes in the intensity and significance of its association with the outcome variable after the change of exposure indicators, and to confirm the consistency of the conclusions under different quantitative methods. Second one, the obese people before pregnancy were excluded and the confounding effect of this subgroup was eliminated to enhance the credibility and effectiveness of the main analysis conclusions (The results of sensitivity analysis are presented in supplementary documents.).

The adjusted odds ratio (_a_OR), and 95% CI (confidence interval) were calculated. Based on previous studies, maternal age, first production, first gestation, chorionicity, mode of conception and gestation age. All the analyses were performed in SPSS software, version 27.0, forest map was drawn in the programs R 4.31, all *P* values were two-tailed, and *P* values < 0.05 were considered significant.

## Results

**Figure 1:**
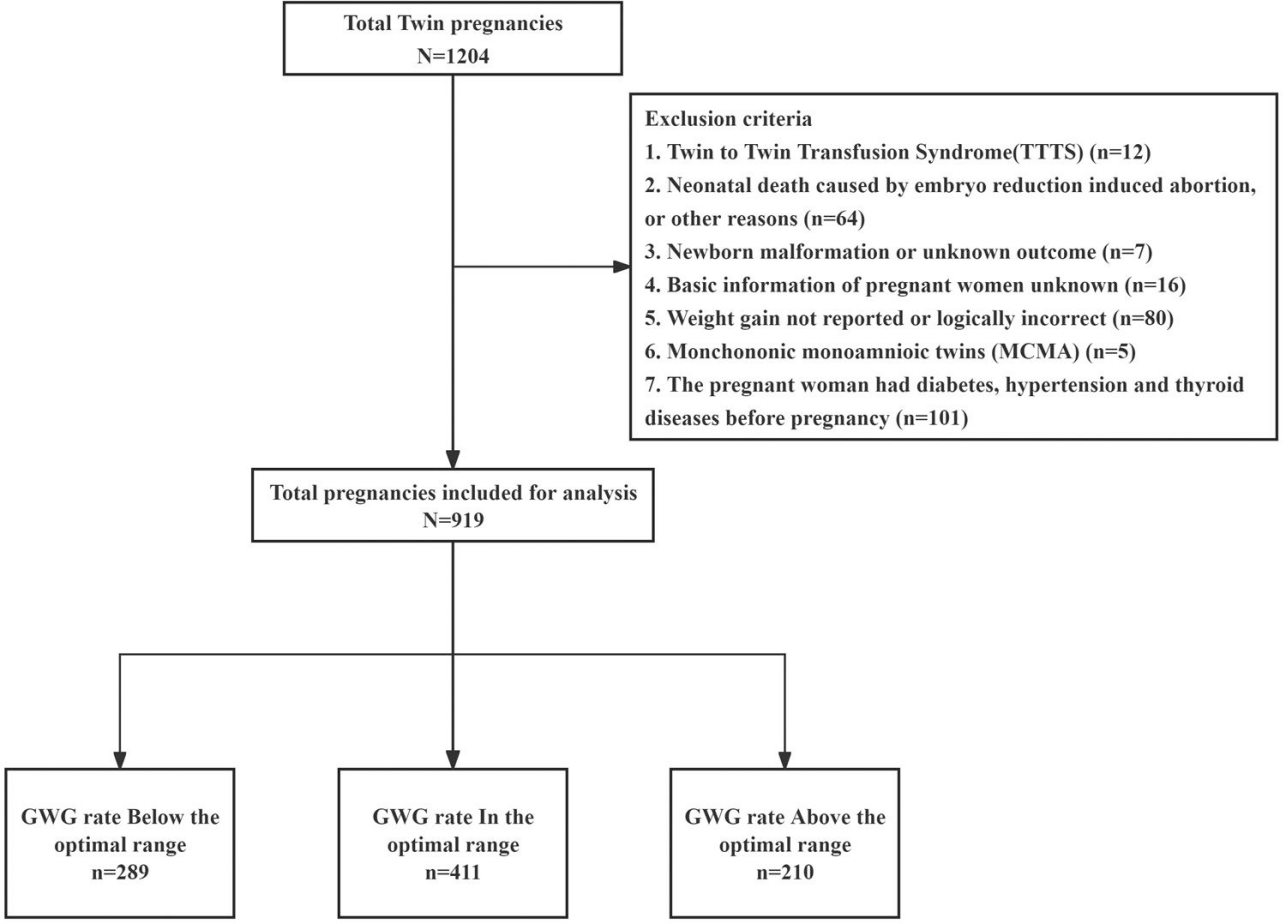
Flowchart of the study.

A total of 1,204 women with twin pregnancies gave birth at the Hunan Maternal and Child Health Hospital. After ruling out the pregnancy records that inconformity to the inclusion criteria (*n* = 285), eventually enrolled 919 twin pregnant women and 1,838 infants were analyzed (Figure 1).

### Maternal outcomes

Of all twin pregnant women,There were 441(44.7%) pregnant women whose GWG rate was in the recommended range, 298(32.4%) pregnant women whose GWG rate was below the recommended range, and 210(22.9%) pregnant women whose GWG rate was above the recommended range. The overall average age of eligible twin pregnant women was 30.78 ± 3.98, 70.6% pregnant women were primiparas, 22.9% pregnant women had monochorionic twin pregnancies, 59.5% of pregnant women became pregnant through ART and 72.3% had normal BMI before pregnancy.There were no significant differences between the three groups for maternal age, first pregnancy, first gestation, chorionicity, mode of conception and pre-pregnancy BMI (*P* > 0.05) (Table 1).

**Table 1:** Characteristics of twin pregnancies by the recommended range of GWG rate.

Characteristics		Below the recommended range 298 (%)	In the recommended range 411 (%)	Above the recommended range 210 (%)	Total group 919 (%)	*P*-value
Maternal age (years)		30.88 ± 4.16	30.71 ± 3.97	30.77 ± 3.77	30.78 ± 3.98	0.856
Maternal age (years) ≥ 35						
	Yes	44 (14.8)	61 (14.8)	33 (15.7)	138 (15.1)	0.949
	No	254 (85.2)	350 (85.2)	177 (84.3)	781 (84.9)	
First pregnancy						
	Yes	149 (50.0)	185 (45.0)	126 (60.0)	460 (50.0)	0.081
	No	149 (50.0)	226 (55.0)	84 (40.1)	459 (50.0)	
First gestation						
	Yes	222 (74.5)	282 (68.6)	145 (69.0)	649 (70.6)	0.201
	No	76 (25.5)	129 (31.4)	65 (31.0)	270 (29.4)	
Chorionicity						
	MC	63 (21.1)	90 (21.9)	48 (22.9)	201 (21.9)	0.899
	DC	235 (78.9)	321 (78.1)	162 (77.1)	718 (78.1)	
Mode of conception						
	Natural pregnancy	120 (40.3)	170 (41.4)	85 (40.5)	375 (40.8)	0.952
	ATR	178 (59.7)	241 (58.6)	125 (59.5)	544 (59.2)	
Pre-pregnancy BMI						
	Underweight	38 (12.8)	49 (11.9)	24 (11.4)	111 (12.1)	0.388
	Normal weight	224 (75.2)	292 (71.0)	149 (71.0)	665 (72.3)	
	Overweight	36(12.0)	70(17.0)	37(17.6)	143(15.6)	

MC, monochorionic; DC, dichorionic; ART, assisted reproductive technology; BMI, body mass index.

A comparison of perinatal maternal outcomes among three groups of pregnant women with twins in different GWG rate categories is presented in Table 2. Compared with the adequate and excessive GWG rate group, the inadequate GWG rate group exhibited a higher rates of preterm birth <37 wk (*P* = 0.022), preterm birth <36 wk (*P* = 0.002), preterm birth <34 wk (*P* < 0.001) and GDM (*P* = 0.017). Conversely, the excessive GWG rate group showed higher rates of subclinical hypothyroidism during pregnancy (*P* = 0.026) and HDP (*P* < 0.001) compared to the inadequate and adequate GWG rate groups. There was no statistically significant difference in the incidence of anemia during pregnancy among the three groups (*P* > 0.05).

**Table 2:** Maternal outcomes grouped by the recommended range of GWG rate.

Outcomes	Below the recommended range 298(%)	In the recommended range 411(%)	Above the recommended range 210(%)	*P*-value
Preterm birth <37 wk	166 (55.7)	187 (45.5)	110 (52.4)	0.022*
Preterm birth <36 wk	96 (32.2)	87 (21.2)	47 (22.4)	0.002*
Preterm birth <34 wk	48 (16.1)	29 (7.1)	16 (7.6)	<0.001*
Subclinical hypothyroidism during pregnancy	69 (23.2)	80 (19.5)	61 (29.0)	0.026*
GDM	95 (31.9)	103 (25.1)	44 (21.1)	0.017*
Anemia during pregnancy	153 (51.3)	195 (47.4)	118 (56.2)	0.115
HDP	56 (18.8)	77 (18.7)	81(38.6)	<0.001*

GDM Gestational Diabetes Mellitus, HDP Hypertensive Disorders of Pregnancy;.

* *P* < 0.05.

**Figure 2:**
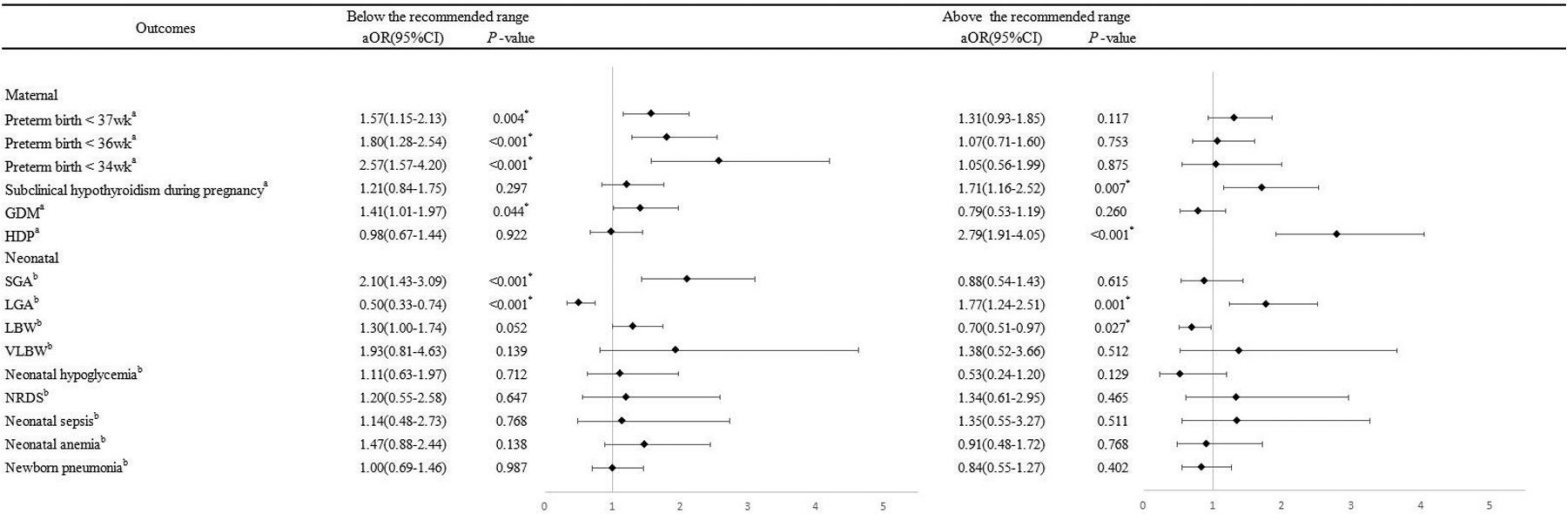
Multivariable logistic analysis of associations between perinatal outcomes and GWG rate. GDM, gestational diabetes mellitus; HDP, hypertensive disorders of pregnancy; SGA, small for gestational age infant; LGA, large for gestational age infant; LBW, low birth weight; VLBW, very low birth weight; NRDS, neonatal respiratory distress syndrome; ^a^Logistic regression model, adjusted for maternal age, first production, first gestation, chorionicity and mode of conception; ^b^Logistic regression model based on the Generalized Estimating Equation, adjusted for maternal age, first production, first gestation, chorionicity, mode of conception and gestation age; *P < 0.05.

In the multivariable logistic regression model, after controlling for potential confounding factors (Figure 2), we observed an increased risk of subclinical hypothyroidism during pregnancy (_a_OR = 1.71, 95% CI:1.16–2.52) and HDP (_a_OR = 2.60,95% CI:1.78–3.78) in pregnant women who had excessive GWG rate compared to those with appropriate. Oppositely, women with inadequate GWG rate were more likely to experience preterm birth (<37 wk: _a_OR = 1.57, 95% CI: 1.15–2.13; <36 wk: _a_OR = 1.80, 95%CI: 1.28–2.54; <34 wk: _a_OR = 2.57, 95%CI:1.57–4.20) and GDM (_a_OR = 1.41, 95%CI1.01–1.97).

### Neonatal outcomes

The outcomes of the newborns are shown in Table 3. Apart from neonatal hypoglycemia (*P* > 0.05), there were statistically significant differences among the three groups of twin pregnant women in terms of SGA (*P* < 0.001), LGA (*P* < 0.001), LBW (*P* < 0.001), VLBW (*P* < 0.001), NRDS (*P* < 0.001), neonatal sepsis (*P* = 0.003), neonatal anemia (*P* < 0.001) and newborn pneumonia (*P* = 0.002). After adjusting for confounding factors (Figure 2), the logistic results of the generalized estimating equation showed that the inadequate GWG increased the risk of SGA (_a_OR = 2.10, 95% CI: 1.43–3.09) and decreased the risk of LGA (_a_OR = 0.50, 95%CI:0.33–0.74), while the excessive GWG rate increased the risk of LGA (_a_OR = 1.77, 95% CI: 1.24–2.51) and also decreased the risk of LBW (_a_OR = 0.50, 95% CI:0.33–0.74).

**Table 3:** Neonatal outcomes grouped by the recommended range of GWG rate.

Neonatal outcomes	Below the recommended range 596(%)	In the recommended range 822(%)	Above the recommended range 420(%)	*P*-value
SGA	82 (13.7)	53 (6.4)	25 (6.0)	<0.001*
LGA	38 (6.4)	104 (12.7)	84 (20.0)	<0.001*
Birth weight < 2,500 g	339 (56.9)	359 (43.7)	169 (40.2)	<0.001*
Birth weight < 1,500 g	38 (6.4)	14 (1.7)	10 (2.4)	<0.001*
Neonatal hypoglycemia	27 (4.5)	32 (3.9)	9 (2.1)	0.129
NRDS	54 (9.1)	33 (4.0)	23 (5.5)	<0.001*
Neonatal sepsis	30 (5.0)	16 (1.9)	11 (2.6)	0.003*
Neonatal anemia	78 (13.1)	48 (5.8)	25 (6.0)	<0.001*
Newborn pneumonia	168 (28.2)	175(21.3)	85(20.0)	0.002*

SGA, small for gestational age infant; LGA, Large for gestational age infant; LBW, low birth weight; VLBW, very low birth weight; NRDS, neonatal respiratory distress syndrome;.

* *P* < 0.05.

### Outcomes according to different GWG rate groups after stratifying pre-pregnancy BMI

Finally, after confounder adjustment and pre-pregnancy BMI stratification, the association between GWG rate and adverse pregnancy outcomes was assessed (Table 4). In the underweight group, the inadequate GWG rate elevated risks of preterm birth <37 wk (_a_OR = 2.61, 95% CI: 1.03–6.63), preterm birth <36 wk (_a_OR = 3.34, 95% CI: 1.19–9.58), and birth weight <2,500 g (_a_OR = 2.28, 95% CI: 1.01–5.18). In contrast, the excessive GWG rate increased preterm birth <36 wk (aOR = 3.34, 95% CI: 1.06–10.55) and HDP (aOR = 4.68, 95% CI: 1.44–15.17) risks.In the normal weight group, the inadequate GWG rate was linked to elevated risks of preterm birth <37 wk (_a_OR = 1.51, 95% CI: 1.05–2.16), preterm birth <36 wk (_a_OR = 1.53, 95% CI: 1.01–2.31), preterm birth <34 wk (_a_OR = 2.58, 95% CI: 1.45–4.59) and SGA (_a_OR = 2.04, 95% CI: 1.29–3.23), but reduced a risk of LGA (_a_OR = 0.40, 95% CI: 0.25–0.65). Conversely, the excessive GWG rate significantly increased the risks of HDP (_a_OR = 3.08, 95% CI: 1.95–4.85) and LGA (_a_OR = 1.85, 95% CI: 1.23–2.76), while significantly decreasing LBG (_a_OR = 0.61, 95% CI:0.41–0.90) risk. In the overweight group, the inadequate GWG rate raised preterm birth <36 wk (_a_OR = 2.96, 95% CI: 1.23–7.10), preterm birth <34 wk (_a_OR = 5.08, 95% CI: 1.12–23.09) and SGA (_a_OR = 4.51, 95% CI: 1.71–11.88) risks, and the excessive GWG rat was associated with subclinical hypothyroidism (_a_OR = 2.75, 95% CI: 1.12–6.74). Across all BMI categories, no statistically significant association was observed between GWG rate and the incidence of GDM.

**Table 4:** Adjusted odds ratios for associations between GWG rate and outcomes stratified by pre-pregnancy BMI levels.

Level	Preterm birth <37 wk	Preterm birth <36 wk	Preterm birth <34 wk	subclinical hypothyroidism	GDM	HDP	SGA	LGA	LBW
	Adjusted Odds ratio (95% CI)^a^	Adjusted Odds ratio (95% CI)^a^	Adjusted Odds ratio (95% CI)^a^	Adjusted Odds ratio (95% CI)^a^	Adjusted Odds ratio (95% CI)^a^	Adjusted Odds ratio (95% CI)^a^	Adjusted Odds ratio (95% CI)^b^	Adjusted Odds ratio (95% CI)^b^	Adjusted Odds ratio (95% CI)^b^
Underweight									
Below the range	2.61 (1.03–6.63)*	3.34 (1.19–9.58)*	1.35 (0.31–5.93)	1.03 (0.34–3.12)	2.35 (0.85–6.46)	0.80 (0.23–2.74)	1.07 (0.36–3.16)	0.64 (0.20–2.00)	2.28 (1.01–5.18)*
Within the range	1.00 (Reference)								
Above the range	2.48 (0.85–7.20)	3.34 (1.06–10.55)*	0.78 (0.12–4.96)	2.13 (0.67–6.79)	1.57 (0.47–5.27)	4.68 (1.44–15.17)*	0.52 (0.13–2.18)	0.97 (0.26–3.67)	0.49 (0.20–1.21)
Normal weight									
Below the range	1.51 (1.05–2.16)*	1.53 (1.01–2.31)*	2.58 (1.45–4.59)*	1.23 (0.80–1.89)	1.26 (0.85–1.87)	0.96 (0.60–1.54)	2.04 (1.29–3.23)*	0.40 (0.25–0.65)*	1.12 (0.80–1.57)
Within the range	1.00 (Reference)								
Above the range	1.24 (0.82–1.87)	0.87 (0.52–1.44)	0.90 (0.41–1.98)	1.42 (0.89–2.28)	0.73 (0.45–1.19)	3.08 (1.95–4.85)*	0.98 (0.56–1.71)	1.85 (1.23–2.76)*	0.61 (0.41–0.90)*
Overweight									
Below the range	1.54 (0.65–3.66)	2.96 (1.23–7.10)*	5.08 (1.12–23.09)*	1.37 (0.52–3.63)	1.75 (0.73–4.19)	1.47 (0.61–3.56)	4.51 (1.71–11.88)*	1.03 (0.40–2.61)	0.17 (0.86–3.37)
Within the range	1.00 (Reference)								
Above the range	1.07 (0.47–2.46)	0.99 (0.38–2.56)	2.19 (0.39–12.40)	2.75 (1.12–6.74)*	0.64 (0.24–1.68)	1.50 (0.63–3.56)	0.67(0.18–2.54)	1.9(0.75–4.79)	1.30(0.65–2.60)

HDP, hypertensive disorders of pregnancy; SGA, small for gestational age infant; LGA, large for gestational age infant.

* *P* < 0.05.

a Logistic regression model, adiusted for maternal age, first production, first gestation, chorionicity and mode of conception.

b Logistic regression model based on the Generalized Estimating Equation, adiusted for maternal age, first production, first gestation, chorionicity; mode of conception and gestation age.

## Discussion

In the overall study population, inadequate gestational weight gain was associated with an increased risk of preterm birth defined at <37 wk (_a_OR = 1.57, 95% CI: 1.15–2.13), <36 wk (_a_OR = 1.80, 95% CI: 1.28–2.54), and <34 wk (_a_OR = 2.57, 95% CI: 1.57–4.20) of gestation, which was supported by the antecedent findings (20, 21). Notably, this association remained robust across sensitivity analyses (Supplementary Figure S1). Currently, the precise mechanism through which insufficient GWG influences the occurrence of preterm birth remains incompletely comprehension. Lin et al. (22) have indicated that women with insufficient GWG may be at an increased risk of pregnancy-related anemia. Pécheux et al. (23) proposed that chronic nutrient deficiency and pregnancy anemia can result in placental dysfunction, giving rise to the stimulation of stress hormone production and ultimately promoting uterine contractions. Furthermore, lack of specific nutrients, such as zinc and copper (24), which might contribute to the premature rupture of membranes or amniotic sac infection, and vitamin deficiency could affect the synthesis of collagen fibers and elastic fibers in fetal membranes, reducing their tensile capacity and increasing the risk of premature membrane rupture (25), which easily increased the chance of premature birth. After stratification by pre-pregnancy BMI, substantial heterogeneity in these associations was observed across BMI categories. This attenuation may be partly explained by reduced statistical power due to smaller sample sizes and fewer outcome events in the overweight group.

The excessive GWG was revealed to be associated with the HDP, which is consistent with previous research findings (26, 27) and sensitivity analyses yielded broadly comparable results (Supplementary Figure S1). As yet, the pathogenesis of HDP due to pre-pregnancy overweight and excessive GWG remains undefined. It has been reported that excessive GWG leads to the accumulation of fat, which in turn results in abnormal lipid metabolism and resistance to leptin and insulin, and can trigger changes in the secretion of signaling molecules and hormones such as TNF and IL-6, ultimately exacerbating cardiovascular diseases (28). No neglecting of the unique pathological changes during pregnancy, including enhanced maternal oxidative stress and impaired endothelial cell function, caused endothelial dysfunction (29) and promote arterial sclerosis. The aforementioned factors make it more likely for twin pregnant women with pre-pregnancy overweight and excessive GWG to develop HDP. While we did not observe an increased risk of HDP in women with excessive GWG who were overweight before pregnancy (_a_OR = 1.50, 95% CI: 0.63–3.56), we did find that being overweight before pregnancy led to a boosted risk of developing HDP (_a_OR = 1.83, 95% CI: 1.23–2.73, *P* = 0.003, Supplementary Table S1). In our opinion, the main reason for this situation was the limited number of samples of overweight and obese pregnant women, which leads to statistically insignificant differences. In comparison to pregnant women with insufficient or normal pre-pregnancy weight, overweight and obese pregnant women were significantly more likely to develop pregnancy-induced hypertension and preeclampsia (30). This might narrow the difference in the probability of developing HPD between pregnant women with normal GWG and those with excessive GWG, particularly among those who were overweight before becoming pregnant.

The mid- to late stages of pregnancy are typically periods of rapid maternal weight gain and increased metabolic demand (31). GDM was typically diagnosed at 24–28 weeks of gestation. Once diagnosed, pregnant women usually adopt strict dietary interventions to limit further weight gain (32). In our study, the exposure was the rate of GWG across the entire pregnancy. This may explain our finding that insufficient GWG was associated with an increased risk of GDM (_a_OR = 1.41, 95% CI: 1.01–1.97), which was consistent with previous studies (20). Similarly, although our results showed an association between an excessively rapid GWG rate and subclinical hypothyroidism during pregnancy (_a_OR = 1.71, 95% CI: 1.16–2.52), this association persisted only in the overweight group after stratification by pre-pregnancy BMI. While previous study have reported associations between elevated maternal TSH levels, lower FT4 concentrations, and gestational weight gain (33), most existing evidence focused on the impact of higher pre-pregnancy BMI on the risk of gestational subclinical hypothyroidism (34, 35). Our findings highlighted the importance of considering pre-pregnancy BMI when interpreting the association between GWG and gestational subclinical hypothyroidism. Although a causal relationship between GWG and GDM or subclinical hypothyroidism cannot be established, emphasized the relevance of pre-pregnancy and early gestational weight gain management in twin pregnancies.

Richardson et al. (36) has demonstrated that singleton pregnant women with low body weight were at a heightened risk of SGA. Our study has yielded similar findings (_a_OR = 2.10, 95% CI: 1.43–3.09). Due to the limited intake of nutrients in the mother's body, there has been a reduction in the supply of nutrients to the placenta. Additionally, twin pregnancies could also lead to relative placental insufficiency and restrict fetal growth. These factors combined result in decreased nutrient delivery to the placenta and ultimately affect fetal development. In contrast, excessive GWG may reflect an overabundant nutritional supply accompanied by elevated insulin levels (37), which can stimulate fetal tissue proliferation and adipose accumulation (38), thereby amplifying the tendency toward increased fetal birth weight (39, 40). This was consistent with our findings in the present study (_a_OR = 1.77, 95% CI: 1.24–2.51). Importantly, these associations remained largely robust in sensitivity analyses (Supplementary Figure S2). However, after stratifying by pre-pregnancy BMI levels, similar results could not be obtained, inadequate GWG was not found to be associated with SGA in the underweight group, which is inconsistent with findings from other study (41). This heterogeneity may be explained by the following mechanisms. The placenta serves as the interface between the mother and fetus, mediating the transfer of nutrients and oxygen essential for fetal growth and development (42). In twin gestations, the placenta was further challenged by increased hemodynamic load and intensified competition for nutrients, which may necessitate additional adaptive responses (43, 44). In pregnancies with either low or high pre-pregnancy BMI, placental function may be suboptimal from early gestation (45, 46), potentially limiting its capacity to respond to variations in maternal GWG. Under these conditions, the relationship between GWG and neonatal outcomes may be modified by both pre-pregnancy BMI and twin-specific physiological demands. This effect modification might contribute to the absence of a clearly detectable independent association between GWG rate and pregnancy outcomes in epidemiological analyses. Compared with SGA, low birth weight was an absolute indicator that did not account for gestational age or fetal growth potential. Accordingly, in sensitivity analyses using total GWG as the exposure, low GWG was identified as a risk factor for low birth weight (_a_OR = 1.38, 95% CI: 1.04–1.82, Supplementary Figure S2). In twin pregnancies, the high rate of preterm birth and intensified nutritional competition may have attenuated the association between gestational weight gain and individual birth weight, while excessive gestational weight gain may still have shifted the birth weight distribution upward, reducing the proportion of low birth weight infants.

There were some advantages in our study. At present, there is a notable absence of authoritative weight gain guidelines for twin pregnancy in China, and our research provides a robust scientific foundation for the lack of this field. We adjusted our results with pre-pregnancy BMI while dividing the total GWG by the duration of pregnancy to avoid bias. In addition, We used GEE models to evaluate neonatal outcomes, accounting for the correlation between twins, thereby obtaining more robust and valid effect estimates and corresponding confidence intervals. At the same time, It's unable to avoid, our study had some limitations. On the one hand, Although we collected extensive information on women with twin pregnancies prior to analysis, data on privacy-related factors (such as nutritional status and socioeconomic status) were unavailable in the medical record system and thus could not be adjusted for as covariates, potentially leading to residual confounding. On the other hand, we utilized total GWG rate as the exposure factor., however, the amount of weight gained during pregnancy varied across the first, second, and third trimesters. Therefore, we were unable to determine during which specific period of twin pregnancy abnormal weight gain was most strongly linked to adverse pregnancy outcomes. In the future, we will conduct prospective research and continuously expand the sample size to investigate additional outcomes, thereby enhancing the epidemiological evidence regarding weight gain during pregnancy and adverse maternal and infant outcomes in twin pregnancies. If circumstances allow, we also aim to conduct our own research on the optimal range of weight gain during pregnancy, in order to further provide guidance and attention to the health of twin mothers and infants.

## Conclusion

In twin pregnancies, inadequate GWG may be highly correlated with preterm birth and SGA. Conversely, excessive GWG might be highly associated with HDP and LGA. During pregnancy, weight gain is a modifiable risk factor that can significantly impact pregnancy outcomes. Therefore, we propose that personalized counseling and guidance on weight gain and nutrition should be initiated before conception and during early pregnancy, and maintained throughout the entire gestational period for women with twin pregnancies, in order to reduce the risk of adverse maternal and neonatal outcomes.

## Data Availability

The datasets generated and analyzed during the current study are not publicly available due to the sensitivity of certain data but are available from the corresponding author on reasonable request.
