# Supplementary material for: Association of gestational weight gain with adverse pregnancy outcomes across body mass index categories for twins in China
Source: Front Reprod Health. 2026 May 14;8:1752008. doi: 10.3389/frph.2026.1752008 (PMC13216204; doi:10.3389/frph.2026.1752008)
Supplement: Supplementary file 2 [file Table1.docx]

**Definitions and diagnostic criteria for secondary outcomes**

**Subclinical hypothyroidism during pregnancy**

Subclinical hypothyroidism during pregnancy was defined as an elevated serum thyroid-stimulating hormone (TSH) level with a normal free thyroxine (FT4) concentration. Specifically, this included women with TSH levels above the pregnancy-specific upper reference limit (or TSH >4.0 mU/L in early pregnancy) with normal FT4, as well as those with TSH levels between 2.5 mU/L and the pregnancy-specific upper reference limit (or < 4.0 mU/L in early pregnancy) with normal FT4.

(Guidelines for prevention and management of thyroid diseases during pregnancy and perinatal period (2022), China)

**Gestational Diabetes Mellitus (GDM)**

Gestational diabetes was diagnosed among pregnant women at 24–28 weeks of gestation using a 75g oral glucose tolerance test, defined by any of the following: fasting blood glucose ≥ 5.1mmol/L, 1-hour post-load glucose ≥ 10.0mmol/L, or 2-hour post-load glucose ≥ 8.5 mmol/L.

(Guideline of diagnosis and treatment of hyperglycemia in pregnancy (2022), China).

**Very Low Birth Weight (VLBW)**

Birth weight less than 1500 g

**Neonatal hypoglycemia**

Neonatal hypoglycemia was defined as a neonatal plasma glucose level below 2.6 mmol/L

(Canadian Paediatric Society.The screening and management of newborns at risk for low blood glucose.Paediatrics & Child Health, 2020)

**Neonatal Respiratory Distress Syndrome (NRDS)**

Neonatal Respiratory Distress Syndrome (NRDS) was diagnosed based on a combination of clinical signs and radiographic findings consistent with NRDS. This included the development of progressive respiratory distress shortly after birth (typically within 6–12 h) characterized by tachypnea, expiratory grunting, nasal flaring, and chest retractions, along with chest radiograph evidence of ground-glass opacities, air bronchograms, or diffuse bilateral involvement. Other causes of respiratory distress (e.g., transient tachypnea of the newborn, congenital lung anomalies, or infection) were excluded.

(According to the guidelines of joint certification of ESPR and UENPS.)

**Neonatal sepsis**

Neonatal sepsis was diagnosed according to Chinese expert consensus criteria, incorporating clinical manifestations, perinatal risk factors, and laboratory findings. Sepsis was excluded in neonates without clinical symptoms, with negative blood cultures, and fewer than two positive nonspecific laboratory markers on two consecutive tests 24 hours apart. Clinical sepsis was diagnosed in symptomatic neonates with either ≥2 positive nonspecific laboratory indicators, cerebrospinal fluid findings consistent with purulent meningitis, or detection of pathogenic DNA in blood. Confirmed sepsis was defined by the presence of clinical signs together with positive cultures from blood, cerebrospinal fluid, or other sterile body fluids.

(Expert consensus on the diagnosis and management of neonatal sepsis (version 2019), China)

**Neonatal anemia:**

Neonatal anemia was diagnosed according to standards described in authoritative domestic clinical textbooks, with comprehensive consideration of hemoglobin levels in relation to postnatal age and gestational age–specific reference ranges. In general, during the early neonatal period (≤1 week after birth), a venous hemoglobin level ≤145 g/L was considered indicative of neonatal anemia, in conjunction with clinical manifestations.

(Practical Pediatric Hematology,China)

**Neonatal pneumonia**

Neonatal pneumonia was diagnosed according to the Chinese expert consensus criteria, based on the presence of respiratory symptoms (such as tachypnea, respiratory distress, grunting, or cyanosis) together with radiographic evidence of pulmonary infiltrates or consolidation on chest X-ray or lung ultrasound. Laboratory indicators of infection and pathogen detection were used as supportive evidence when available.

(Pneumonia. Semin Fetal Neonatal Med.)

All twin neonates were evaluated by neonatologists after birth. Preterm infants and those presenting with respiratory distress or other clinical concerns were directly admitted to the neonatal unit for observation and treatment. Neonatal anemia was assessed based on routine postnatal laboratory testing. Neonates without apparent abnormalities were also examined daily by neonatologists during routine postnatal care, and were promptly referred to the neonatal unit if any clinical issues were identified. All diagnoses were made by attending neonatologists based on standard clinical criteria. Diagnostic testing was performed as clinically indicated rather than through universal screening.

| **Supplement Table1 Multivariable logistic analysis of associations between perinatal outcomes and BMI.** | | | | | | | |
| --- | --- | --- | --- | --- | --- | --- | --- |
| Outcomes | Underweight |  | *P*-value |  | Overweight |  | *P*-value |
| Maternal |  |  |  |  |  |  |  |
| Preterm birth <37wk^a^ | 0.76(0.50-1.16) |  | 0.204 |  | 1.65(1.13-2.40) |  | 0.009^*^ |
| Preterm birth <36wk^a^ | 1.38(0.87-2.18) |  | 0.170 |  | 1.70(1.14-2.56) |  | 0.010^*^ |
| Preterm birth <34wk^a^ | 1.08(0.57-2.07) |  | 0.807 |  | 0.97(0.51-1.83) |  | 0.924 |
| Subclinical hypothyroidism during pregnancy^a^ | 0.99(0.60-1.63) |  | 0.983 |  | 1.20(0.79-1.83) |  | 0.394 |
| GDM^a^ | 1.18(0.74-1.89) |  | 0.485 |  | 1.20(0.80-1.79) |  | 0.375 |
| HDP^a^ | 0.94(0.57-1.55) |  | 0.799 |  | 1.83(1.23-2.73) |  | 0.003^*^ |
| Neonatal |  |  |  |  |  |  |  |
| SGA^b^ | 0.95(0.56-1.60) |  | 0.841 |  | 1.12(0.59-2.15) |  | 0.724 |
| LGA^b^ | 1.58(0.90-2.75) |  | 0.110 |  | 1.28(0.67-2.46) |  | 0.455 |
| LBW^b^ | 0.84(0.58-1.21) |  | 0.350 |  | 0.80(0.50-1.26) |  | 0.337 |
| Abbreviations: GDM gestational diabetes mellitus, HDP hypertensive disorders of pregnancy, SGA small for gestational age infant, LGA large for gestational age infant, LBW low birth weight, VLBW very low birth weight, NRDS neonatal respiratory distress syndrome;  ^a^ Logistic regression model, adiusted for maternal age, first production, first gestation, chorionicity and mode of conception;  ^b^ Logistic regression model based on the Generalized Estimating Equation , adiusted for maternal age, first production, first gestation, chorionicity; mode of conception and gestation age;  ^*^ *P* < 0.05 | | | | | | | |
|  | | | | | | | |
